# High serum copper as a risk factor of all-cause and cause-specific mortality among US adults, NHANES 2011–2014

**DOI:** 10.3389/fcvm.2024.1340968

**Published:** 2024-04-19

**Authors:** Xianghui Zeng, Lanqian Zhou, Qingfeng Zeng, Hengqing Zhu, Jianping Luo

**Affiliations:** ^1^ Department of Cardiology, Ganzhou Hospital of Traditional Chinese Medicine, Ganzhou, Jiangxi, China; ^2^ Department of Anesthesiology, The Second Affiliated Hospital of Nanchang University, Nanchang, Jiangxi, China; ^3^ Emergency Department, The Second Affiliated Hospital of Gannan Medical University, Ganzhou, Jiangxi, China; ^4^ Department of Cardiology, Ganzhou Hospital of Guangdong Provincial People’s Hospital, Ganzhou Municipal Hospital, Ganzhou, Jiangxi, China; ^5^ Department of Cardiology, Ganzhou People’s Hospital, Ganzhou, Jiangxi, China

**Keywords:** serum copper, all-cause mortality, cancer mortality, cardiovascular disease mortality, national health and nutrition examination survey

## Abstract

**Background:**

Several studies have shown that serum copper levels are related to coronary heart disease, diabetes, and cancer. However, the association of serum copper levels with all-cause, cause-specific [including cardiovascular disease (CVD) and cancer] mortality remains unclear.

**Objectives:**

This study aimed to prospectively examine the association of copper exposure with all-cause, CVD, and cancer mortality among US adults.

**Methods:**

The data for this analysis was obtained from the National Health and Nutrition Examination Survey (NHANES) between 2011 and 2014. Mortality from all-causes, CVD, and cancer mortality was linked to US National Death Index mortality data. Cox regression models were used to estimate the association between serum copper levels and all-cause, CVD, and cancer mortality.

**Results:**

A total of 2,863 adults were included in the main study. During the mean follow-up time of 81.2 months, 236 deaths were documented, including 68 deaths from cardiovascular disease and 57 deaths from cancer. The weighted mean overall serum copper levels was 117.2 ug/L. After adjusting for all of the covariates, compared with participants with low (1st tertile, <103 μg/L)/medium (2st tertile, 103–124 μg/L) serum copper levels, participants with high serum copper levels (3rd tertile, ≥124 μg/L) had a 1.75-fold (95% CI, 1.05–2.92)/1.78-fold (1.19,2.69) increase in all-cause mortality, a 2.35-fold (95% CI, 1.04–5.31)/3.84-fold (2.09,7.05) increase in CVD mortality and a 0.97-fold (95% CI, 0.28–3.29)/0.86-fold (0.34,2.13) increase in cancer mortality. In addition, there was a linear dose-response association between serum copper concentration with all-cause and CVD mortality (*P* for nonlinear > 0.05).

**Conclusions:**

This prospective study found that serum copper concentrations were linearly associated with all-cause and CVD mortality in US adults. High serum copper levels is a risk factor for all-cause and CVD mortality.

## 1 Introduction

Cardiovascular disease (CVD) mortality is one of the leading causes of death worldwide (1). The economic burden of CVD and related mortality in the United States is particularly troubling (2). CVD mortality is usually attributed to physical inactivity, obesity, atherosclerosis, hypertension, and diabetes (3, 4). The imbalance of certain metallic elements in the body also contributes (5–7). Metal elements are exposed more and more frequently in our daily lives and can be ingested in a variety of ways, including air inhalation, skin contact, and ingestion of metal-contaminated food (8–10). Recent study evidence suggests that metal deficiency or excess in the body may adversely affect human health (11–16).

Copper, an essential trace element, has played a vital role in biochemical processes and physiological regulation in all forms of life (17, 18). Copper can participate in physiological functions such as oxidative phosphorylation, angiogenesis, blood coagulation, and anti-oxidation (17). As a cofactor for various enzymes, copper also plays an in dispensable in biochemical processes such as erythropoiesis, cellular respiration, cholesterol, and glucose metabolism, pigment formation, and hormone synthesis (18, 19). Serum copper homeostasis is very important to human health and is tightly regulated. Both serum copper deficiency (<63.7 ug/L) and excess (>140.12 ug/L) can have harmful effects that can lead to serious illness (11). Monk's disease and Wilson's disease are caused by serum copper deficiency (20). A retrospective cohort study involving 183 patients with cirrhosis or portal hypertension found that serum copper deficiency was an independent risk factor for death in patients with advanced liver disease (21). Serum copper levels were significantly lower in patients who died after COVID-19 infection than in the healthy population. Serum copper excess can generate reactive oxygen species that cause oxidative damage to lipids, proteins, and other molecules, increasing the risk of atrial fibrillation and heart failure (22, 23). Many epidemiological studies have confirmed the association between excessive serum copper exposure and impairments of renal function, lung function, and neurological function (24–26). High serum copper levels were found to increase the risk of CVD mortality in men in a previous small sample study (27). In patients with lung cancer, serum copper levels were positively associated with all-cause mortality (28). However, in the Turkish study, serum copper levels were not associated with all-cause mortality risk in patients with sepsis and systemic inflammatory response syndrome (29). Although these studies in small samples or subgroups of the population have suggested that lack of or excessive serum copper exposure may have some association with mortality, the correlation between serum copper levels and all-cause, CVD, and cancer mortality in a nationally representative general population remains unclear.

Notably, current research data on the association of serum copper levels with all-cause, CVD, and cancer mortality is limited. Therefore, the purpose of this study was to assess the association between serum copper levels and all-cause and cause-specific mortality in US adults using data from the National Health and Nutrition Examination Survey (NHANES).

## 2 Methods

### 2.1 Study population

Data for the current study has been abstracted from the NHANES database, which is aimed at investigating the fitness and nutrient composition of the US population. The survey is conducted through the National Center for health information. All the participants or their proxies provided written informed consent. The survey was approved by the Institutional Review Board of the United States Centers for Disease Control and Prevention (30). NHANES is a publicly available database. In this study, a total of 11,539 participants aged ≥18 years were included from NHANES 2011–2014. Excluding serum copper deficiency (*n* = 7,931) and failure to follow up (*n* = 4), 3,604 participants were included in the sensitivity analysis. After removing 741 participants with missing covariates, 2,863 participants were included in the final model analysis (Supplementary Figures S1, S2).

### 2.2 Assessment of serum copper

Blood samples were drawn in a fasted state and transported to the laboratory for analysis under suitable freezing (–20°C conditions). Serum copper was measured using inductively coupled plasma–dynamic reaction cell–mass spectrometry in a trace element clean laboratory. The lower limit of detection (LLOD) was 2.5 ug/dl for NHANES (2011–2014) samples. For serum copper concentrations below the LLOD, the value was replaced with a value equal to the limit of detection divided by the square root of 2. More details about the laboratory analysis strategy can be found on the NHANES official website (31).

### 2.3 Mortality ascertainment

The outcomes of this study included all-cause mortality, and cause-specific (CVD and cancer) mortality, which were ascertained by linkage to the National Death Index through December 31, 2019. The document linked NHANES to the national dying index through a rigorous opportunity matching and death certificate overview procedure. All-cause mortality was defined as death from any cause. Cause-specific mortality was defined using the International Classification of Diseases (ICD), 10th Revision (ICD-10) codes for potential causes of death for cardiovascular disease (ICD-10 codes 053-075) and cancer (ICD-10 codes 019-043) (32). Follow-up time was calculated as the interval between the date of the serum copper examination and the date of demise, or the end of follow-up, whichever befell earlier.

### 2.4 Covariates

Self-reported demographic information (age, sex, education, race); health behaviors (alcohol intake, smoking); and diagnoses of diabetes, coronary heart disease, stroke, cancer, hypertension, chronic obstructive pulmonary disease, and hyperlipidemia were collected. Body weight and height were measured to calculate Body mass index (BMI). Self-reported race or ethnicity using fixed categories was collected to characterize the population. The race was classified as non-Hispanic, white, and other. Education levels were categorized as less than high school or high school and over. The smoking status was subdivided into nonsmokers, former smokers, and current smokers, including daily and often. Hypertension was defined as systolic blood pressure (SBP) ≥ 140 mmHg, diastolic blood pressure (DBP) ≥ 90 mmHg, or taking antihypertensive agents. Diabetes was defined as a fasting blood glucose level ≥126 mg/dl, non-fasting blood glucose level ≥200 mg/dl, self-reported history of diabetes, or taking glucose-lowering medications. BMI was calculated as weight in kilograms divided by height in meters squared. The estimated glomerular filtration rate (eGFR, ml/min/1.73 m^2^) was calculated using the Chronic Kidney Disease Epidemiology equation (2021). Total cholesterol and high-density lipoprotein (HDL) cholesterol were assessed under a standardized process and protocol (33, 34).

## 3 Statistical analysis

All analyses were performed using SAS version 9.4 (SAS Institute). Two-sided *P* < 0.05 was considered statistically significant. The overall sampling design and sampling weights were adjusted to account for non-response bias and sampling design using the PROC SURVEY (SVY) commands in SAS (35).

The weighted continuous and categorical variables were expressed using means (95%CI) and frequencies (%). Continuous variables were analyzed using ANOVA (36), while those with categorical variables were analyzed using the Chi-square test. Participants' serum copper levels were divided into three equal groups, low (<103 ug/L), medium (103–123 ug/L), and high (≥124 ug/L). Kaplan-Meier curves have been used to estimate the survival probability of serum copper level tertiles. The Cox proportional-hazard regression model was used to estimate hazard ratios (HRs) and 95% confidence intervals (CIs) for the association between serum copper exposure and all-cause, cardiovascular disease, and cancer mortality. We constructed two models. Model 1 was adjusted for covariates, which included age, sex, race, and education levels; model 2 was adjusted for model 1 plus BMI, smoking status, hypertension, diabetes, eGFR, HDL cholesterol, and total cholesterol. The linear trend was tested by a median value for each tertile as a continuous variable. We investigated the nonlinear association between serum copper and mortality by including restricted cubic spline curves with nodes at the 10th, 50th, and 90th quartiles in the fully adjusted model. We also examined the association between serum copper levels and all-cause and cardiovascular disease mortality by performing analyses stratified by age (<60 and ≥60 years), sex, and race (non-Hispanic white or other). The interplay between non-stop serum copper and stratification becomes tested. Considering the exclusion of 741 participants with missing covariates, we used multiple interpolations for further analysis of 3,604 participants to assess the robustness of our results. Multiple interpolations were performed using Surveyimpute and MIANALYZE methods (37).

## 4 Results

A total of 2,863 participants were enrolled in the main analysis and during the mean follow-up time of 81.2 months, 236 deaths were documented, including 68 deaths from cardiovascular disease and 57 deaths from cancer. The weighted mean overall serum copper concentration was 117.2 ug/L. The numbers of Serum copper deficiency (<63.7 ug/L), normal ranges (63.7–140.12 ug/L), and copper excesses (>140.12 ug/L) were observed in 25, 2,861, and 718, respectively (Supplementary Table S1). According to the tertiles of serum copper levels, participants were divided into low, medium, and high serum copper levels groups (respectively, serum copper concentration < 103 ug/L, 103 ug/L ≤ serum copper concentration < 124 ug/L and serum copper concentration ≥ 124 ug/L). Compared with participants with low serum copper levels, participants with higher serum copper levels were more likely to be female, older, of other races, have a high annual family income, former smokers, have antihypertensive medications, hypertension, and have a higher BMI, as well as elevated total cholesterol and HDL cholesterol (Table 1).

**Table 1:** Baseline characteristics of participants by serum copper levels among 2,863 participants in NHANES (2011–2014).

Characteristic	No. (%)				
	All	Serum copper levels (μg/L)			
		Low (<103)	Middle (103–123)	High (≥124)	*P* value
Number of participants^a^	*N* = 2,863	*N* = 898	*N* = 934	*N* = 1,031	
Serum copper, mean (95% CI)	117.2 (114.75,119.7)	90.3 (89.4,91.19)	112.7 (112.24,113.24)	148.6 (145.92,151.38)	<.0001
Age, years, mean (95% CI)	47.8 (46.73,48.82)	45.1 (43.63,46.47)	49.4 (47.79,50.97)	48.8 (47.13,50.53)	<.0001
Sex					<.0001
Male	1,455 (49.9)	688 (77.2)	518 (52.2)	249 (20.5)	
Female	1,408 (50.1)	210 (22.8)	416 (47.8)	782 (79.5)	
Races/ethnicity					<.0001
Non-Hispanic White	1,230 (68.6)	403 (71.3)	429 (71.4)	398 (63.2)	
Non-Hispanic Black	628 (10.4)	124 (5.7)	191 (9.1)	313 (16.3)	
Mexican American	325 (8.1)	91 (7.9)	107 (8.1)	127 (8.3)	
Other Race	680 (12.9)	280 (15.1)	207 (11.4)	193 (12.2)	
Education levels					0.0778
Less than high school	605 (14.5)	152 (12.2)	211 (15.4)	242 (15.9)	
More high school than	2,258 (85.5)	746 (87.8)	723 (84.6)	789 (84.1)	
Annual family income					<.0001
Under $20,000	701 (17.4)	180 (13.9)	212 (14.6)	309 (23.7)	
Over $20,000	2,162 (82.6)	718 (86.1)	722 (85.4)	722 (76.3)	
Smoking status					0.0004
Never	1,618 (56)	546 (61)	480 (49.8)	592 (57.5)	
Former	686 (24.7)	208 (23.5)	268 (29.3)	210 (21.1)	
Now	559 (19.3)	144 (15.5)	186 (20.8)	229 (21.3)	
Alcohol consumption					0.1565
Never	428 (11.3)	114 (10)	134 (10.2)	180 (13.6)	
Former	462 (13.8)	136 (12.9)	161 (14.4)	165 (14)	
Now	1,973 (74.9)	648 (77.1)	639 (75.4)	686 (72.4)	
Medications use					
Lipid-lowering medications^d^	595 (20.1)	176 (20)	214 (21.6)	205 (18.7)	0.5305
Hypoglycemic medications^c^	321 (9)	84 (8.2)	97 (8.3)	140 (10.6)	0.3411
Antihypertensive medications^b^	197 (6.2)	34 (3.3)	72 (7.3)	91 (7.8)	0.0002
Baseline comorbidities					
Coronary heart disease	105 (3.3)	31 (3.2)	40 (3.6)	34 (3.1)	0.8711
Stroke	97 (2.4)	24 (1.9)	23 (1.9)	50 (3.4)	0.1142
COPD	126 (5)	21 (3.1)	46 (6.3)	59 (5.6)	0.0118
Hypertension	1,215 (38.8)	312 (33.3)	414 (40.8)	489 (42.2)	0.0105
Diabetes	527 (14.4)	137 (12.4)	149 (13.3)	241 (17.6)	0.0211
Hyperlipidemia	1,993 (68.9)	560 (62.4)	668 (71.2)	765 (73)	0.0043
Cancer	297 (9.8)	79 (9.3)	119 (11.8)	99 (8.4)	0.1387
BMI, kg/m^2^, mean (95% CI)	28.9 (28.47,29.24)	27 (26.55,27.48)	28.8 (28.33,29.33)	30.7 (30.01,31.43)	<.0001
Biochemical tests, mean (95% CI)					
EGFR, ml/min per 1.73 m^2^	93.7 (92.58,94.8)	93.7 (91.73,95.72)	93.3 (91.52,95.05)	94.1 (92.33,95.8)	0.5684
Total cholesterol, mmol/L	5 (4.93,5.03)	4.8 (4.68,4.92)	5 (4.93,5.1)	5.1 (5.05,5.23)	<.0001
HDL cholesterol, mmol/L	1.4 (1.35,1.4)	1.3 (1.26,1.33)	1.4 (1.36,1.43)	1.4 (1.4,1.48)	<.0001
Follow-up time, months, mean (95% CI)	81.2 (79.19,83.16)	81.7 (79.42,84.05)	81.8 (79.58,84.09)	79.9 (77.52,82.36)	0.0011
Cause of death					
All-cause mortality	236 (7.2)	60 (5.3)	70 (6.6)	106(9.7)	0.0135
Cardiovascular mortality	68(1.8)	19(1.3)	15(1)	34(3.2)	0.0062
Cancer mortality	57(2)	13(1.6)	19(2.5)	25(2)	0.5279

NHANES, national health, and nutrition examination survey; COPD, chronic obstructive pulmonary disease; BMI, body mass index; HDL, high-density lipoprotein; eGFR, estimated glomerular ﬁltration rate; CI, confidence interval.

Percentages and means (95% CI) were estimated using US population weights.

*P* values were computed separately for each covariate and indicate statistically significant differences between step groups if *P* < 0.05.

Race/ethnicity was determined using preferred terminology from the National Center for Health Statistics as non-Hispanic white, non-Hispanic black, Mexican American, and Other Race.

^a^ Numbers in the table were unweighted.

^b^ Antihypertensive medications referred to self-reported taking antihypertensive drugs in NHANES.

^c^ Hypoglycemic medications included oral hypoglycemic agents and treatment with insulin (participant self-report).

^d^ Lipid-lowering medications included atorvastatin, simvastatin, pravastatin, rosuvastatin, lovastatin, and pitavastatin. fluvastatin, niacin, and ezetimibe.

The association between serum copper exposure and all-cause, CVD, and cancer mortality was demonstrated (Table 2 and Supplementary Table S2). In Model 1, HRs for all-cause mortality, CVD mortality, and cancer mortality in participants with medium/high serum copper levels compared with those with low serum copper concentrations were 1.88 (95% CI, 1.17–3.03)/2.06 (1.37,3.08), 2.81 (95% CI, 1.16–6.79)/4.72 (2.66,8.4), and 1.00 (95% CI, 0.33–3.05)/0.95 (0.39,2.3), respectively. The full-adjusted HRs (Model 2) for all-cause, CVD, and cancer mortality were 1.75 (95% CI, 1.05–2.92)/1.78(1.19,2.69), 2.35 (95% CI, 1.04–5.31)/3.84(2.09,7.05), and 0.97 (95% CI, 0.28–3.29)/0.86(0.34,2.13) for participants with high serum copper level compared with those with low/medium serum copper concentrations, respectively. In addition, for per SD (30 ug/L), increase in serum copper concentration, all-cause and CVD mortality risk increased 1.53 times (95% CI, 1.02–2.30), and 1.32 times (95% CI, 1.04–1.69), respectively, after the adjustment for all covariates. However, the serum copper was not associated with cancer mortality [HR: 1.01 (95% CI–0.53,1.9)].

**Table 2:** Hazard ratios (95% CIs) of all-cause, cardiovascular disease and cancer cortality according to serum copper levels among 2,863 participants in NHANES (2011–2014).

Serum copper levels	Patients, No.^a^	Events, No.^a^	Mortality rate per 1,000 person-years	Model 1	Model 2
All-cause mortality					
Low	898	60	8.1	1 (ref)	1 (ref)
Middle	934	70	9.9	0.92 (0.60,1.40)	0.98 (0.63,1.54)
High	1,031	106	14.8	1.88 (1.17,3.03)	1.75 (1.05,2.92)
*P* for trend				0.0049	0.0179
Serum copper, per 30 μg/L	2,863	236	10.7	1.38 (1.1,1.72)	1.32 (1.04,1.69)
Cardiovascular disease mortality					
Low	898	19	2	1 (ref)	1 (ref)
Middle	934	15	1.6	0.60 (0.28,1.29)	0.61 (0.29,1.28)
High	1,031	34	5.3	2.81 (1.16,6.79)	2.35 (1.04,5.31)
*P* for trend				0.0086	0.0151
Serum copper, per 30 μg/L	2,863	68	2.8	1.57 (1.10,2.23)	1.53 (1.02,2.30)
Cancer mortality					
Low	898	13	2.6	1 (ref)	1 (ref)
Middle	934	19	3.8	1.06 (0.39,2.82)	1.13 (0.40,3.16)
High	1,031	25	3.1	1.00 (0.33,3.05)	0.97 (0.28,3.29)
*P* for trend				0.9823	0.912
Serum copper, per 30 μg/L	2,863	57	3	0.97 (0.56,1.7)	1.01(0.53,1.9)

Model 1 was adjusted for age, sex, race, and education levels, annual family income, smoking status, alcohol consumption.

Model 2 was adjusted for the variables in model 1 plus lipid-lowering medications, hypoglycemic medications, antihypertensive medications, stroke, COPD, hypertension, hyperlipidemia, diabetes, BMI, eGFR, total cholesterol, and HDL cholesterol.

NHANES, national health, and nutrition examination survey; CI, confidence interval; COPD, chronic obstructive pulmonary disease; BMI, body mass index; HDL, high-density lipoprotein; eGFR, estimated glomerular ﬁltration rate.

^a^ Numbers in the table were unweighted.

**Figure 1:**
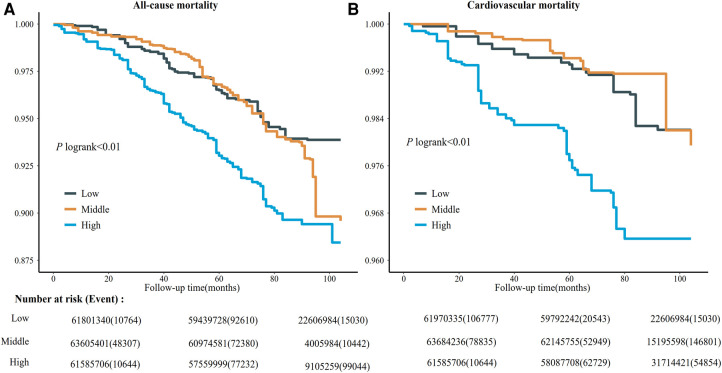
Kaplan-meier survival curve for all-cause (**A**) and cardiovascular disease mortality (**B**) among different levels of serum copper concentration. The survival rate was evaluated after adjusting for the variables for age, sex, race, education levels, annual family income, smoking status, alcohol consumption, lipid-lowering medications, hypoglycemic medications, antihypertensive medications, stroke, COPD, hypertension, hyperlipidemia, diabetes, BMI, eGFR, total cholesterol, and HDL cholesterol. The number at risk (event) was estimated using US population weights. NHANES, national health, and nutrition examination survey; CI, confidence interval; COPD, chronic obstructive pulmonary disease; BMI, body mass index; HDL, high-density lipoprotein; eGFR, estimated glomerular ﬁltration rate. Participants were subdivided into three subgroups according to serum copper levels: low, serum copper <103 μg/L; middle, 103 μg/L ≤ serum copper <124 μg/L; high, serum copper ≥124 μg/L.

**Figure 2:**
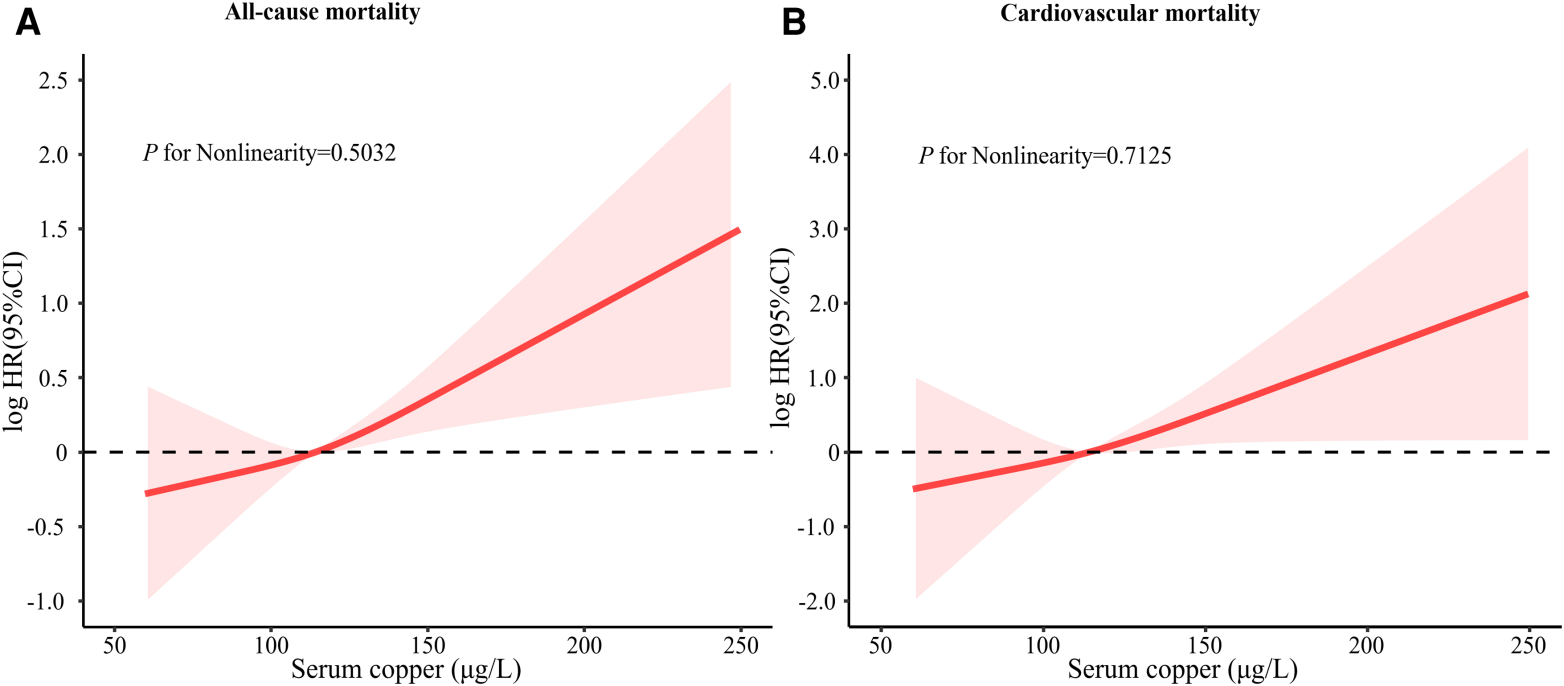
Restricted cubic spline of log HR of serum copper concentration with CVD mortality and all-cause mortality. In a fully adjusted model, restricted cubic spline curves with sections at the 10th, 50th, and 90th quartiles were added to examine the nonlinear relationship between serum copper concentration with CVD mortality and all-cause mortality. The model was adjusted for age, sex, race, education levels, annual family income, smoking status, alcohol consumption, lipid-lowering medications, hypoglycemic medications, antihypertensive medications, stroke, COPD, hypertension, hyperlipidemia, diabetes, BMI, eGFR, total cholesterol, and HDL cholesterol. NHANES, national health, and nutrition examination survey; CI, confidence interval; COPD, chronic obstructive pulmonary disease; BMI, body mass index; HDL, high-density lipoprotein; eGFR, estimated glomerular ﬁltration rate.

Kaplan–Meier curves of all-cause and risk for the serum copper tertiles were presented in Figure 1. Participants with high serum copper concentration had higher all-cause and CVD mortality risk than those with low serum copper concentration. After multivariate adjustment, there was a linear dose-response association between serum copper concentration with CVD mortality and all-cause mortality (*p* for nonlinear > 0.05) (Figure 2).

The stratified analysis according to age (<60 years, 60 years≤), sex, race (Non-Hispanic White, Other Race), or BMI (<30 kg/m^2^, 30 kg/m^2^≤) were given in Table 3. There was no evidence that the association between serum copper levels and all-cause and CVD mortality differed by race, age, or sex.

**Table 3:** Stratified analyses of the associations between serum copper levels and all-cause mortality or cardiovascular disease mortality among 2,863 participants in NHANES (2011–2014).

	Serum copper levels			
Characteristic	Low	Middle	High	*P*- interaction
All-cause mortality				
Age, year				0.3564
<60	1 (ref)	0.79 (0.26,2.42)	1.92 (0.62,5.91)	
60≤	1 (ref)	1.03 (0.63,1.7)	1.63 (0.87,3.06)	
Sex				0.0702
Male	1 (ref)	1.14 (0.68,1.91)	2.25 (1.22,4.14)	
Female	1 (ref)	0.52 (0.22,1.23)	0.96 (0.43,2.14)	
Races/ethnicity				0.1524
Non-Hispanic White	1 (ref)	1.02 (0.59,1.76)	1.97 (1.06,3.67)	
Other Race	1 (ref)	0.74 (0.34,1.63)	1.3 (0.71,2.4)	
BMI, kg/m^2^				0.8777
<30	1 (ref)	0.87 (0.49,1.53)	1.6 (0.92,2.79)	
30≤	1 (ref)	1.18 (0.52,2.66)	2.26 (0.82,6.2)	
Cardiovascular disease mortality				
Age, year				0.1939
<60	1 (ref)	0.26 (0,33.73)	2.15 (0.06,78.6)	
60≤	1 (ref)	0.6 (0.3,1.19)	1.75 (0.77,3.98)	
Sex				0.1428
Male	1 (ref)	0.43 (0.12,1.5)	3.13 (1.34,7.29)	
Female	1 (ref)	0.46 (0.09,2.28)	1.16 (0.3,4.5)	
Races/ethnicity				0.4752
Non-Hispanic White	1 (ref)	0.41 (0.15,1.12)	2.47 (1.05,5.8)	
Other Race	1 (ref)	2.17 (0.63,7.48)	3.03 (0.96,9.59)	
BMI, kg/m^2^				0.413
<30	1 (ref)	0.36 (0.1,1.24)	1.46 (0.45,4.79)	
30≤	1 (ref)	1.68 (0.52,5.44)	6.36(1.21,33.32)	

COX proportional hazards models were used to estimate HRs (95% CI) for all-cause and cardiovascular disease mortality based on serum copper levels. Results were adjusted for age, sex, race, educations levels, annual family income, smoking status, alcohol consumption, lipid-lowering medications, hypoglycemic medications, antihypertensive medications, stroke, COPD, hypertension, hyperlipidemia, diabetes, BMI, eGFR, total cholesterol, HDL cholesterol, except for the sub-group variable.

NHANES, national health, and nutrition examination survey; HR, hazard ratio; CI, confidence interval; COPD, chronic obstructive pulmonary disease; BMI, body mass index; HDL, high-density lipoprotein; eGFR, estimated glomerular ﬁltration rate.

To assess potential bias from missing values, we used multiple imputations to impute the missing values of the covariates (*n* = 741) and then repeated the analysis. The analysis was consistent with the results of the main analysis (Supplementary Table S3). After adjusting for all of the covariates, compared with the low serum copper group, participants with high serum copper levels had a 1.72-fold (95% CI, 1.13–2.62) increase in all-cause mortality, a 2.36-fold (95% CI, 1.09–5.11) increase in CVD mortality, respectively. HRs for cancer mortality were 1.07 (0.43,2.66)/0.98 (0.37,2.6) for participants with medium/high serum copper levels compared to individuals with low serum copper concentrations, respectively.

## 5 Discussion

### 5.1 Major findings

In this large prospective cohort study, we investigated the association between serum copper exposure and all-cause, CVD, and cancer mortality in US adults. Our study found that higher serum copper concentrations in US adults were associated with increased all-cause and CVD mortality after adjusting for important potential confounders, but not cancer mortality. The study provides new evidence for the association of copper exposure with all-cause and CVD mortality in US adults. These results are noteworthy because serum copper concentrations have gotten little attention for many years.

### 5.2 Comparisons with previous studies

This study suggests that higher serum copper concentration is associated with higher CVD mortality in US adults. We also found higher serum copper levels in patients with hypertension, diabetes, and hyperlipidemia (Table 1), implying that higher serum copper levels are associated with mortality from systemic metabolic diseases/disorders. Several previous studies have supported our findings. Elevated serum copper levels have been reported to increase heart failure by affecting systolic and diastolic blood pressure functions (38). The oxidation of low-density lipoprotein cholesterol is exacerbated by copper, causing atherosclerosis (39) and increasing the risk of cardiovascular disease (40).In addition, high serum copper concentrations lead to insulin resistance and abnormal insulin secretion, which promotes diabetes (41, 42). In a study of 3,253 Germans undergoing coronary angiography, elevated serum copper concentrations were associated with an increased risk of death from cardiovascular causes (43). Also, after reviewing 37 studies, Chowdhury et al. found that higher circulating copper concentrations were associated with an increased risk of cardiovascular disease (44). On the other hand, copper binds to homocysteine to form a copper-homocysteine complex, which leads to vascular dysfunction and increases the risk of cardiovascular disease (45).

At the same time, our study also found that higher serum copper concentrations in US adults were associated with increased all-cause mortality. A longitudinal study of elderly Italians showed a positive association between serum copper concentrations and all-cause mortality (46). Similarly, a study of 167 lung cancer patients showed that higher serum copper levels were associated with increased all-cause mortality in lung cancer patients (28), and in a study of 498 elderly patients, Malavolta et al. (47) indicated that elevated plasma copper led to increased plasma copper/zinc ratio in patients with cardiovascular disease and that copper/zinc ratio was an important predictor of all-cause mortality in the elderly. The above studies are consistent with our results.

Interestingly, our study found no significant effect of high or low serum copper levels on the risk of cancer mortality in the general U.S. adult population. Studies have demonstrated that the risk of colorectal and breast cancers is not associated with serum copper (48, 49). However, high serum copper levels may increase the risk of lung cancer (50). Higher serum copper levels may be associated with decreased survival in patients with liver and lung cancers (28, 51). These findings indicate that serum copper has been differentially associated with different cancers, but in the general population, serum copper is not associated with the risk of cancer death. In conclusion, the link between copper and cancer is complicated and calls for more investigation into the underlying mechanisms.

### 5.3 Underlying mechanism

However, the underlying mechanism by which higher blood copper concentrations increase all-cause mortality remains unclear. Some studies suggest that copper may play a role in diabetes (52), dyslipidemia (53, 54), and cancer (55), exacerbating all-cause mortality. In a Japanese study, higher copper intake was strongly associated with an increased risk of type 2 diabetes, and the association was more pronounced in overweight and smokers (52). Galiardi et al. (53) found that high serum copper levels increase cholesterol levels, leading to dyslipidemia. A study in NHANES (2011–2014) revealed that high serum copper concentrations were associated with elevated total serum cholesterol concentrations and increased risk of dyslipidemia (54). In addition, a Chinese study demonstrated that high serum copper levels significantly increased the risk of oral cancer (55). Excessive serum copper increases the risk of cell death. A novel form of cell death, copper-dependent, has recently been reported to cause proteotoxic stress and cell death through copper binding to the thiooctylated components of the tricarboxylic acid cycle (56). Although serum copper is an essential micronutrient, excess copper can produce reactive oxygen radicals that lead to oxidative stress (57). When copper binds to superoxide dismutase 1, it increases the level of reactive oxygen species, which in turn causes oxidative stress (58, 59). In addition, copper may also be involved in inflammatory processes, with high levels of copper promoting reactive oxygen species formation and participating in inflammation (60).

### 5.4 Strengths and limitations

This study has many strengths. Strengths of the current study include a prospective study design, a relatively large sample size, and the use of a nationally representative sample of US adults, which help generalize our findings. In addition, with the detailed and high-quality data collection in the NHANES, this study was able to control potential confounding effects.

Our study has several limitations. First, the number of deaths from cardiovascular and cancer in this study was small, which may have limited the statistical power to detect a significant association. Second, in baseline data, the number of cancer patients was relatively small, so it was impossible to analyze the association between the mortality rate of cancer patients and blood copper level. In the end, we get death results from death certificates, which may contain inaccurate information—some causes of death will be miscoded and may not generalize to non-fatal events. Therefore, our study could only provide a limited view of the effects of copper on cardiovascular health.

## 6 Conclusions

In summary, this nationally representative cohort study revealed that serum copper concentrations were linearly associated with all-cause and CVD mortality. High serum copper levels is a risk factor for all-cause and CVD mortality, and not cancer mortality. Future studies of all-cause and CVD mortality should take serum copper concentrations into account.

## Data Availability

Publicly available datasets were analyzed in this study. This data can be found here: NHANES database: https://www.cdc.gov/nchs/nhanes/index.htm.
