# Supplementary material for: High serum copper as a risk factor of all-cause and cause-specific mortality among US adults, NHANES 2011–2014
Source: Front Cardiovasc Med. 2024 Apr 19;11:1340968. doi: 10.3389/fcvm.2024.1340968 (PMC11066204; doi:10.3389/fcvm.2024.1340968)
Supplement: Supplementary file 1 [file Datasheet1.pdf]

Supplementary Material

High Serum Copper as predictors of All-cause and Cause-Specific Mortality Among US Adults, NHANES 2011–2014

Xianghui Zeng, PhD[1#]; Lanqian Zhou[2#]; Qingfeng Zeng[1,3]; Hengqing Zhu[4]; Jianping Luo, PhD[5*]

[1]Department of Cardiology, Ganzhou Hospital of Traditional Chinese Medicine, Ganzhou, Jiangxi, China;

[2]Department of Anesthesiology, The Second Affiliated Hospital of Nanchang University, Nanchang, Jiangxi, China;

[3]Emergency Department, The Second Affiliated Hospital of Gannan Medical University, Ganzhou, Jiangxi, China;

[4]Department of Cardiology, Ganzhou Hospital of Guangdong Provincial People's Hospital, Ganzhou Municipal Hospital, Ganzhou, Jiangxi, China;

[5]Department of Cardiology, Ganzhou People's Hospital, Ganzhou, Jiangxi, China.

#Contributed equally

*** Correspondence: Corresponding Author:** Jianping Luo

Mail to: Ganzhou People's Hospital, 17 Hongqi Dadao, Ganzhou, Jiangxi, China.

E-mail: luojianping@mail.gzsrmyy.com

1 Supplemental Table

Supplemental Table 1. Characteristics of the participant serum copper level.

Supplemental Table 2. Hazard ratios (95% CIs) of all-cause, cardiovascular disease and cancer mortality according to serum copper levels among 2863 participants in NHANES (2011-2014).

Supplemental Table 3. Hazard ratios (95% CIs) of all-cause, cardiovascular disease, and cancer mortality according to serum copper levels using surveyimpute for multiple interpolation (n=3604).

2 Supplemental Figure

Supplemental Figure 1. Number and percentage of missing values of covariates.

Supplemental Figure 2. Flowchart of the study participants.

Supplemental Table 1. Characteristics of the participant serum copper level.

**Serum copper levels (μg/L)**	**No.**	**%, weighted**	**95% CI, weighted**
Deficiency (<63.7)	25	0.75	0.39-1.1
Normal range (63.7-140.12)	2861	81	78.8-83.2
Excess (≥140.12)	718	18.2	15.9-20.6

Supplemental Table 2. Hazard ratios (95% CIs) of all-cause, cardiovascular disease and cancer mortality according to serum copper levels among 2863 participants in NHANES (2011-2014).

Serum copper levels	Model 1	*P*	Model 2	*P*
**All-cause mortality**				
Low (<103)	1.09(0.72,1.67)	0.6744	1.02(0.65,1.59)	0.9317
Middle (103-124)	1(ref)		1(ref)	
High (124≤)	2.06(1.37,3.08)	0.001	1.78(1.19,2.69)	0.007
**CVD mortality**				
Low (<103)	1.68(0.78,3.64)	0.1803	1.63(0.78,3.42)	0.1867
Middle (103-124)	1(ref)		1(ref)	
High (124≤)	4.72(2.66,8.4)	<.0001	3.84(2.09,7.05)	<.0001
**Cancer mortality**				
Low (<103)	0.95(0.35,2.54)	0.9129	0.89(0.32,2.48)	0.8122
Middle (103-124)	1(ref)		1(ref)	
High (124≤)	0.95(0.39,2.3)	0.907	0.86(0.34,2.13)	0.7321

Model 1 was adjusted for age, sex, race, and education levels, annual family income, smoking status, alcohol consumption.
Model 2 was adjusted for the variables in model 1 plus lipid-lowering medications, hypoglycemic medications, antihypertensive medications, stroke, COPD, hypertension, hyperlipidemia, diabetes, BMI， eGFR, total cholesterol, and HDL cholesterol.
Abbreviations: NHANES, National Health, and Nutrition Examination Survey; CI, confidence interval; COPD, chronic obstructive pulmonary disease; BMI, body mass index; HDL, high-density lipoprotein; eGFR, estimated glomerular filtration rate.

Supplemental Table 3. Hazard ratios (95% CIs) of all-cause, cardiovascular disease, and cancer mortality according to serum copper levels using surveyimpute for multiple interpolation (n=3604).

Serum copper levels	Patients, No.*	Events, No.*	Mortality rate per 1000 person-years	Model 1	Model 2
**All-cause mortality**					
Low	1149	70	7.8	1(ref)	1(ref)
Middle	1144	86	10	0.93(0.64,1.36)	1.02(0.73,1.44)
High	1311	127	14.3	1.73(1.12,2.66)	1.72(1.13,2.62)
P for trend				0.0043	0.0059
Serum copper, per 30 µg/L	3604	283	10.5	1.34(1.07,1.67)	1.31(1.06,1.63)
**Cardiovascular disease mortality**					
Low	1149	20	1.7	1(ref)	1(ref)
Middle	1144	20	1.8	0.76(0.36,1.58)	0.83(0.39,1.77)
High	1311	37	4.5	2.39(1.04,5.49)	2.36(1.09,5.11)
P for trend				0.0122	0.0081
Serum copper, per 30 µg/L	3604	77	2.5	1.5(1.04,2.17)	1.52(1.07,2.16)
**Cancer mortality**					
Low	1149	15	2.4	1(ref)	1(ref)
Middle	1144	20	3.3	0.99(0.4,2.45)	1.07(0.43,2.66)
High	1311	29	2.9	0.96(0.38,2.47)	0.98(0.37,2.6)
P for trend				0.9354	0.9278
Serum copper, per 30 µg/L	3604	64	2.7	0.98(0.59,1.62)	0.99(0.58,1.71)

* Numbers in the table were unweighted.
Model 1 was adjusted for age, sex, race, and education levels, annual family income, smoking status, alcohol consumption.
Model 2 was adjusted for the variables in model 1 plus lipid-lowering medications, hypoglycemic medications, antihypertensive medications, stroke, COPD, hypertension, hyperlipidemia, diabetes, BMI, eGFR, total cholesterol, and HDL cholesterol.
Abbreviations: NHANES, National Health, and Nutrition Examination Survey; CI, confidence interval; COPD, chronic obstructive pulmonary disease; BMI, body mass index; HDL, high-density lipoprotein; eGFR, estimated glomerular filtration rate.

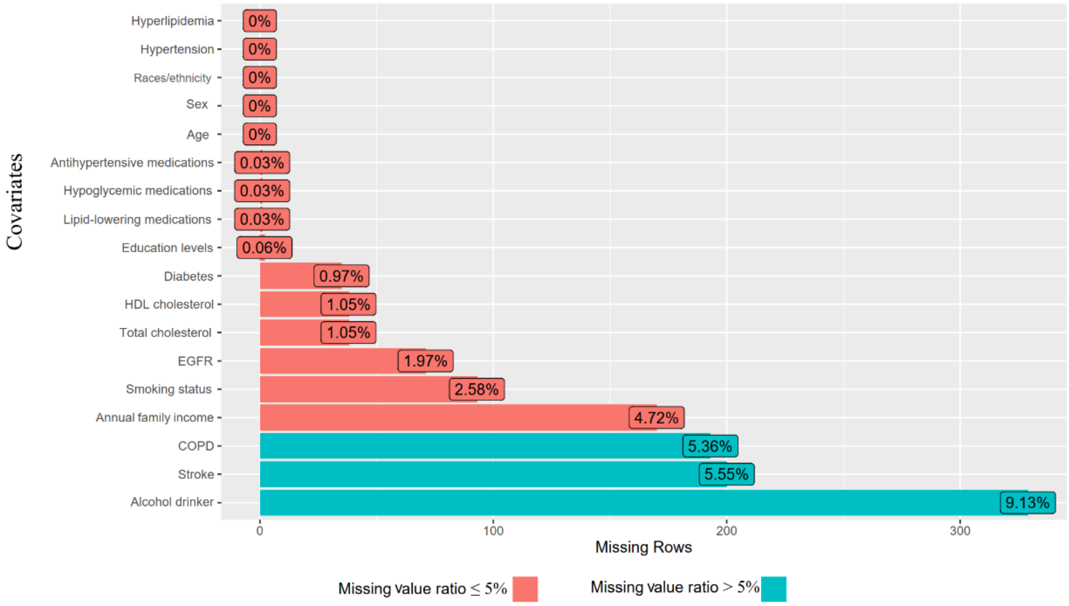

Supplemental Figure 1. Number and percentage of missing values of covariates.

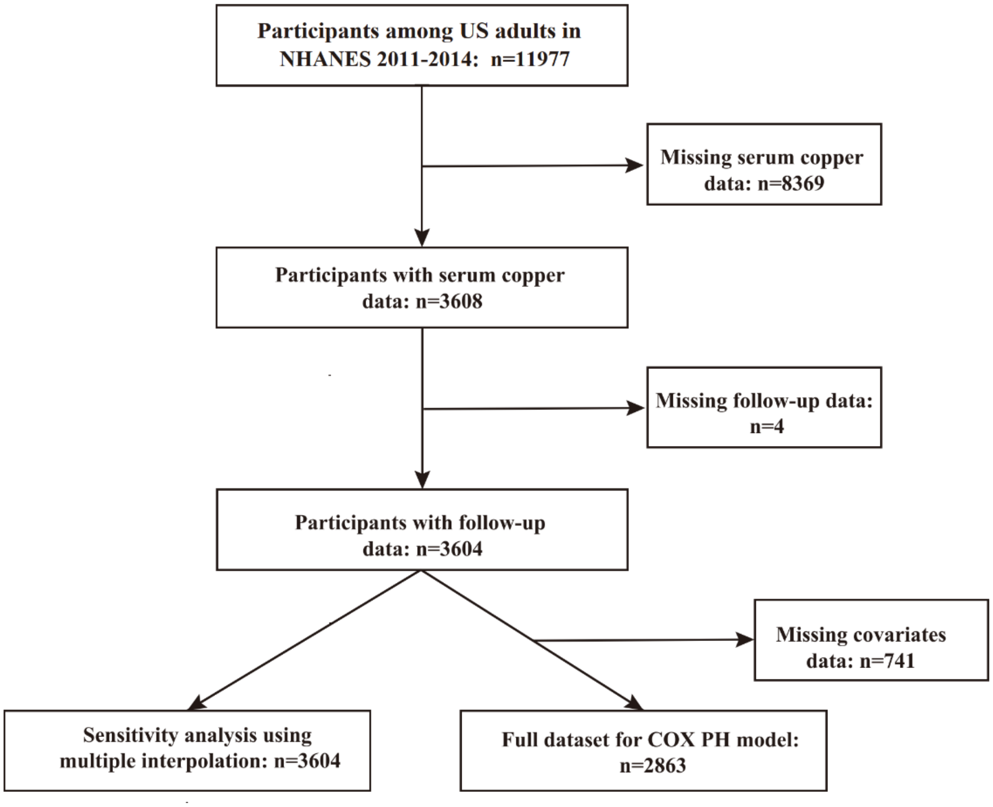

Supplemental Figure 2. Flowchart of the study participants.
